# HIV-1 latency in activated T-cells

**DOI:** 10.1186/1742-4690-10-S1-O22

**Published:** 2013-09-19

**Authors:** Ben Berkhout

**Affiliations:** 1 Academic Medical Center (AMC), University of Amsterdam, Netherlands

## 

HIV-1 latency remains a formidable barrier towards virus eradication as therapeutic attempts to purge these reservoirs are so far unsuccessful. The pool of transcriptionally silent proviruses is established early in infection and persists for a lifetime, even when viral loads are suppressed below detection levels using anti-retroviral therapy. Upon therapy interruption the reservoir can re-establish systemic infection. Different cellular reservoirs that harbor latent provirus have been described. In this study we demonstrate that HIV-1 can also establish a silent integration in actively proliferating primary T lymphocytes. Co-culturing of these proliferating T lymphocytes with dendritic cells (DCs) activated the provirus from latency. Activation did not involve DC-mediated C-type lectin DC-SIGN signaling or TCR-stimulation but was mediated by DC-secreted component(s) and cell-cell interaction between DC and T lymphocyte that could be inhibited by blocking ICAM-1 dependent adhesion. These results imply that circulating DCs could purge HIV-1 from latency and re-initiate virus replication. Moreover, our data show that viral latency can be established early after infection and supports the idea that actively proliferating T lymphocytes with an effector phenotype contribute to the latent viral reservoir. Unraveling this physiologically relevant purging mechanism could provide useful information *for* the development of new therapeutic strategies that aim at the eradication of HIV-1 reservoirs.

